# Enhancing Robustness of Sortase A by Loop Engineering and Backbone Cyclization

**DOI:** 10.1002/chem.202002740

**Published:** 2020-08-18

**Authors:** Zhi Zou, Diana M. Mate, Maximilian Nöth, Felix Jakob, Ulrich Schwaneberg

**Affiliations:** ^1^ Institute of Biotechnology RWTH Aachen University Worringerweg 3 52074 Aachen Germany; ^2^ DWI–Leibniz-Institute for Interactive Materials Forckenbeckstraβe 50 52074 Aachen Germany; ^3^ Current address: Center of Molecular Biology “Severo Ochoa” Universidad Autónoma de Madrid Nicolás Cabrera 1 28049 Madrid Spain

**Keywords:** biocatalysis, protein engineering, site-specificity, sortase A, thermal stability

## Abstract

*Staphylococcus aureus* sortase A (SaSrtA) is widely used for site‐specific protein modifications, but it lacks the robustness for performing bioconjugation reactions at elevated temperatures or in presence of denaturing agents. Loop engineering and subsequent head‐to‐tail backbone cyclization of SaSrtA yielded the cyclized variant CyM6 that has a 7.5 °C increased melting temperature and up to 4.6‐fold increased resistance towards denaturants when compared to the parent rM4. CyM6 gained up to 2.6‐fold (vs. parent rM4) yield of conjugate in ligation of peptide and primary amine under denaturing conditions.

## Graphical abstract

**Protein engineering**: Semi‐rational loop engineering and subsequent head‐to‐tail backbone cyclization of *Staphylococcus aureus* sortase A (SaSrtA) yielded a cyclized variant CyM6 with enhanced activity and robustness towards thermal and chemical denaturation (see figure).

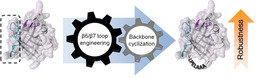

Functionalization of proteins is often performed by targeting reactive endogenous amino acid side chains (e.g. ‐NH_2_ in lysine or ‐SH in cysteine residues).^[1]^ However, in many cases, cysteine is limited as accessible nucleophile of proteins.^[2]^ In contrast, the high abundance of accessible lysine often leads to heterogeneously modified products (e.g. 19 lysine residues are present on the surface of a cellulase).[^3^, ^4^] Bond‐forming enzymes such as sortase,^[5]^ butelase,^[6]^ transglutaminase,^[7]^ lipoic acid ligase,^[8]^ biotin ligase,^[9]^ phosphopantetheinyl transferase,^[10]^ SnoopLigase,^[11]^ and SpyLigase^[12]^ have emerged as powerful tools for site‐specific protein modifications. Sortases are a family of transpeptidases that are found in Gram‐positive bacteria.^[13]^ The mechanism of transpeptidation of *Staphylococcus aureus* sortase A (SaSrtA) is well studied. SaSrtA recognizes protein 1 (P1) which harbors an LPXTG (in which X equals any amino acid) motif and cleaves the amide bond between threonine and glycine. The resulting protein‐sortase thioester is then attacked by the amino group of an N‐terminal glycine residue of protein 2 (P2), resulting in a protein conjugate of protein 1 and protein 2 (Scheme 1).^[14]^

**Scheme 1:**
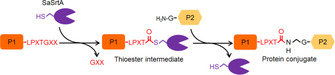
Schematic representation of sortase‐mediated ligation of LPETG‐tagged and GGG‐tagged proteins.

Owing to the high degree of site‐specificity and the minor size (≤5 AA residues) of the sorting motif, sortase‐mediated transpeptidation, also known as sortagging, has become a widely used method for protein conjugation,^[15]^ cyclization,^[16]^ labelling/functionalization,^[17]^ purification^[18]^ and/or immobilization.[^19^, ^20^] Given to the comparably high transpeptidase activity among the sortases,^[21]^ SaSrtA is most frequently used in sortagging applications.^[22]^ However, the overall low activity (catalytic efficiency *k*_cat_/*K*_M_=160 s^−1^ m ^−1^)[^9^, ^23^] and the requirement of calcium as a cofactor^[24]^ of SaSrtA wild type (WT) hinders broader utilization of sortagging. Protein engineering campaigns were performed to yield SaSrtA variants with increased catalytic efficiencies in aqueous solutions (up to 105‐fold),[^9^, ^23^] organic co‐solvents (up to 6.3‐fold),^[25]^ or in absence of calcium cofactor (up to 114‐fold).^[26]^ By employing engineered SaSrtA variants, expanded applications such as reversible surface functionalization,^[27]^ *in vivo* protein modifications,^[28]^ as well as continuous and flow‐based protein immobilization and labelling were achieved.[^29^, ^30^] Notably, the advantages such as low nucleophile concentration and immediate product release in continuous and flow‐based systems highlight the potential of sortagging in industrial applications.^[29]^ The latter further requires robust SaSrtA variants with high process stabilities (e.g. thermal/storage stability, resistance to denaturants) over a long period of time. A few studies reported the engineering of SaSrtA WT for increased stability towards thermal and chemical denaturation (e.g. 11.2 °C improvement in melting temperature (T_m_) and 4.5‐fold resistance in presence of 2.5 m urea).[^31^, ^32^] However, engineering of highly active SaSrtA variants with enhanced tolerance against thermal and chemical stress has not been reported yet.

In this study, a first engineering campaign of the SaSrtA variant rM4 (P94S/D160N/D165A/K196T)^[9]^ towards high thermal stability was performed. SaSrtA rM4 showed significantly higher activity (>75‐fold vs. WT at ambient temperature)[^9^, ^25^] but remarkably low thermal stability (T_m_=48.6 vs. 59.4 °C of WT, Figure S3).^[31]^ In the protein engineering campaign, a high throughput screening (HTS) assay of SaSrtA was employed based on the reconstitution of the self‐sufficient cytochrome P450 BM3 (CYP102A1) monooxygenase from *Bacillus megaterium* (Figure 1 a). The P450 BM3 monooxygenase consists of a heme and a reductase domain, which are fused by a flexible linker region in a single polypeptide chain.^[33]^ We separated the heme and the reductase domain at a flexible linker and tagged the heme domain with the C‐terminal LPETG motif and the reductase domain with the N‐terminal triglycine (Table S1). Heme and reductase domains were individually expressed and purified (Figure S1). By using SaSrtA, the heme and reductase domain are reconstituted into the active and full‐length P450 BM3 monooxygenase, which forms a dimer preventing a back reaction (Figure 1 a).[^34^, ^35^] The amount of reconstituted P450 BM3 was detected by a reported fluorogenic assay using 7‐benzoxy‐3‐carboxycoumarin ethyl ester (BCCE) as the substrate (Figure 1 a).^[36]^ Reconstitution of heme and reductase domain to full‐length P450 BM3 using SaSrtA WT was previously reported.^[37]^ However, the utilization of the P450 BM3 reconstitution in screening of activity improved sortase A has not been reported yet.

**Figure 1:**
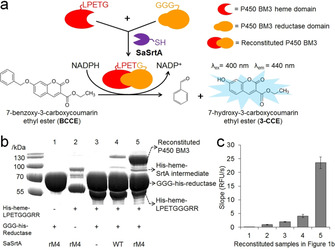
Principle and validation of P450 BM3 reconstitution assay for high‐throughput screening system of SaSrtA. a) Schematic representation of the P450 BM3 reconstitution assay. LPETG‐tagged heme and GGG‐tagged reductase domains were reconstituted to self‐sufficient full‐length P450 BM3. Amounts of reconstituted full‐length P450 were evaluated via a fluorogenic BCCE assay. b) SDS‐PAGE analysis of the splicing of LPETG‐tagged P450 BM3 heme and GGG‐tagged P450 BM3 reductase domains by SaSrtA. c) BCCE assay of domain splicing samples in b. High activity in BCCE assay indicates high activity of SaSrtA in P450 reconstitution.

The generation of full‐length P450 BM3 by SaSrtA rM4 was confirmed by sodium dodecyl sulfate polyacrylamide gel electrophoresis (SDS‐PAGE) (Figure 1 b **/**S2). In comparison to SaSrtA WT, rM4 catalyzed a significantly higher amount of reconstituted full‐length P450 BM3 after sortagging (Figure 1 b/S2). Samples were subsequently employed in the BCCE assay. In sample 3, background activity in BCCE assay was observed (Figure 1 c). It is suggested that the copresence of heme and reductase domains also contributes the intermolecular electron transfer^[33]^ and thus lead to increased activity (vs. samples 1 and 2). SaSrtA rM4 showed a 5.8‐fold improved activity (vs. WT) in the BCCE assay (Figure 1 c). The latter highlights the applicability of the P450 BM3 reconstitution assay to screen for activity improved SaSrtA variants.

The P450 BM3 reconstitution based microtiter plate (MTP) screening system was established and employed to evolve SaSrtA for increased thermal stability (Figure S4/S5). SaSrtA rM4 cell free extracts were incubated at 55 °C for 1 h (15 % of initial activity was retained, Figure S3) before reconstitution of the heme‐LPETGGGRR and GGG‐reductase domain was initiated (Figure S4). A coefficient of variation (CoV) of 17.6 % after thermal incubation (55 °C for 1 h) of SaSrtA rM4 was achieved in the 96‐well MTP (Figure S5). The CoV of rM4 without thermal incubation was 13.5 % (Figure S5). CoVs below 20 % have routinely been used in protein engineering campaigns.[^38^, ^39^, ^40^]

Previous studies revealed that the highly mobile β6/β7 loop (from position 159 to 172, Figure 2 b) of SaSrtA adapts an “open and close” model to recognize the LPXTG sorting motif in the presence of calcium.[^24^, ^41^] Amino acid substitutions in the β6/β7 loop were reported to alter catalytic activity (e.g. D160N, D165A, G167E, or Q172H)[^9^, ^42^] or organic solvent resistance (e.g. R159G, D165Q, D170W).^[25]^ Based on these initial findings, we conducted a structural analysis and identified an array of positions in the β6/β7 loop that might also contribute to enhanced activity and (or) stability of SaSrtA. Aiming to efficiently identify which positions in β6/β7 loop contribute to loop stabilization, eleven single site‐saturation mutagenesis libraries (SSM: 159, 161, 162, 163, 164, 166, 167, 168, 169, 170, and 172) were generated in the β6/β7 loop of SaSrtA rM4 (Table S2), 168 colonies per position were picked and screened with the P450 BM3 reconstitution assay with and without thermal incubation (55 °C for 1 h, Figure S4). SaSrtA variants with 1.25‐fold or higher improved activity (vs. rM4 after thermal incubation) were selected for further rescreening (four replicates each). Finally, five variants with a >1.50‐fold increased activity (Figure 2 a) after thermal incubation (55 °C for 1 h) were identified at three positions 159 (R159N and R159T), 162 (K162N and K162P) and 172 (Q172L). Notably, the amino acid substitution (K162P) in variant rM4 resulted in 3.5‐fold increased activity and 3.1‐fold increased thermal stability (Figure 2 a). The substitution of proline residue (K162P) is expected to reduce the flexibility (increase thermal stability) since its ring limits the conformational mobility of SaSrtA.^[43]^ Thermal stability and catalytic activity are usually contradicting properties (high activity requires flexibility; high thermal stability requires rigidity). However, in this case, surprisingly increased activities of variant rM4‐K162P (3.5‐fold) and rM4‐R159N (3.0‐fold) were observed. Hydrophobic interactions between the leucine reside in the LPXTG motif and β6/β7 loop act as a main factor for substrate binding.^[41]^ The increased activities of rM4‐K162P and rM4‐R159N may attribute to their enhanced hydrophobic interactions toward the LPETG substrate.

**Figure 2:**
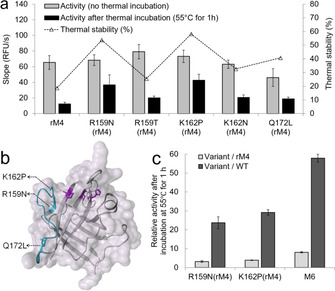
Loop engineering of SaSrtA for improved thermal stability. a) Identified variants in β6/β7 loop after screening of SSM libraries at eleven rationally selected positions. Thermal stability is defined as the ratio (percentage) of activity after thermal incubation (55 °C for 1 h) divided by activity without thermal treatment. b) Structure of SaSrtA (PDB: 1IJA). The three active site residues (His120, Cys184, and Arg197 are marked in magenta, from right to left). The β6/β7 loop was marked in cyan and the identified substitutions (R159N, K162P, and Q172L) are labelled. c) Relative activity of recombined SaSrtA variants after thermal incubation (55 °C for 1 h).

To further enhance the thermal stability of rM4, substitutions R159N was combined with rM4‐K162P and resulted in the variant rM4‐R159N/K162P (hereafter M6). Variant M6 was purified and the activity was determined using the reported fluorescence resonance energy transfer (FRET) assay for sortase characterization.^[44]^ Accumulating the beneficial effects from both K162P and R159N substitutions, variant M6 showed an 8.1‐fold increased activity and 4.6‐fold increased thermal stability (vs. parent rM4; 55 °C for 1 h, Figure 2 c/S6). In comparison to SaSrtA WT, a remarkable 58‐fold increased activity of M6 was observed (Figure 2 c/S6). Unfortunately, an additional recombination attempt of Q172L to M6 resulted in a variant (rM4‐R159N/K162P/Q172L) with reduced activity and stability (Figure S6).

Head‐to‐tail backbone cyclization (HtTBC) has been reported to improve tolerance of proteins (e.g. green fluorescent protein,^[45]^ cytokines^[46]^) towards thermal incubation, protease digestion, or chaotropic agents.^[43]^ HtTBC of SaSrtA WT was previously implemented for enhanced stability against urea using an intein‐mediated posttranslational modification.^[32]^ To further improve stability properties, HtTBC of SaSrtA M6 was performed using a sortase A from *Streptococcus pyogenes* (SpSrtA). SpSrtA recognizes orthogonal sorting motifs (e.g. LPELA, Figure 3 a) which differ from the preferred SaSrtA motif (LPETG).^[21]^

**Figure 3:**
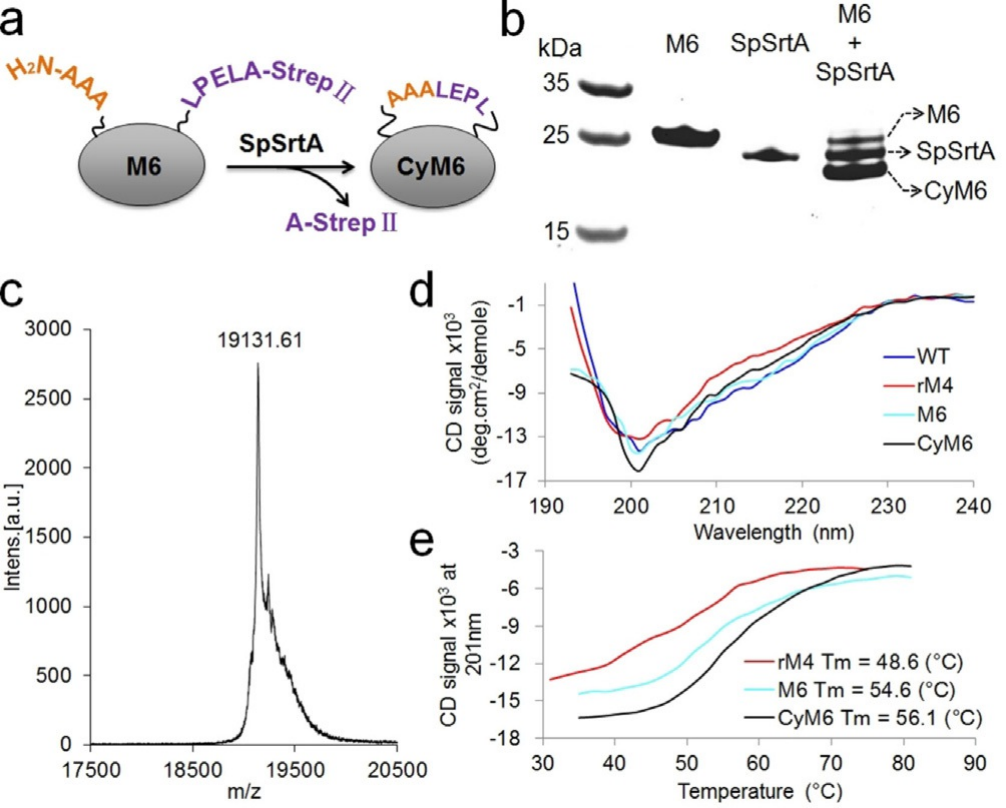
Head‐to‐tail backbone cyclization (HtTBC) of SaSrtA M6. a) Schematic representation of HtTBC of AAA‐SaSrtA M6‐LPELA‐Strep II using *Streptococcus pyogenes* sortase A (SpSrtA). b) Analysis of cyclization of SaSrtA M6 in sodium dodecyl sulfate polyacrylamide gel electrophoresis (SDS‐PAGE). c) Confirmation of the formation of CyM6 by matrix‐assisted laser desorption/ionization mass spectrum (MALDI‐MS). The expected size of CyM6 is 19131.55 Dalton. d) Circular dichroism (CD) spectra of linear and cyclized SaSrtA variants under wavelength from 193 to 240 nm. e) Melting curves of linear and cyclized SaSrtAs. Plots were obtained by recording the CD spectrum signals of SaSrtA at 201 nm under gradient temperatures.

The generation of cyclized SaSrtA M6 (hereafter CyM6) was initially confirmed via SDS‐PAGE. After a six‐hour reaction time, a majority (≥80 %, data is not shown) of SaSrtA M6 was cyclized (Figure 3 b). Upon the cyclization, the C‐terminal Strep II in M6 is cleaved and subsequently removed from the reaction mixture using column based chromatography (Figure S7). The correct mass of CyM6 was determined through matrix‐assisted laser desorption/ionization mass spectrum analysis (MALDI‐MS, Figure 3 c). Owing to the flexible and spatial proximity N‐ and C‐termini of SaSrtA (Figure 2 b), the fusion of termini showed little effect on the overall structural conformation of the enzyme. CyM6 retained 99 % of its specific activity after cyclization when compared to the linear SaSrtA M6 (Figure S8). This result is in agreement with a previous report, showing that SaSrtA WT retained full activity after cyclization.^[32]^

Circular dichroism (CD) spectra revealed no detectable differences between CyM6 and SaSrtA M6, rM4 and WT at ambient temperature (Figure 3 d). The T_m_ of SaSrtA variants were determined by CD spectroscopy under gradient temperatures (from 30 to 80 °C, Figure S9). M6 gains 6.0 °C in T_m_ when compared to the parent rM4 (T_m_ of M6 and rM4 is 54.6 and 48.6 °C, respectively) and interestingly, CyM6 has a further 1.5 °C increased T_m_ compared to M6 (T_m_ of CyM6 is 56.1 °C; Figure 3 e). In summary, the engineered CyM6 shows a remarkably improved thermal stability (ΔT_m_=+7.5 °C) when compared to the starting parent rM4.

Thermal stability of enzymes often goes in hand with storage stability as well as resistance against denaturants.^[45]^ Therefore, storage stability of CyM6 at room temperature was investigated. CyM6 and M6 retained more than 80 % of its initial activity after fourteen days at room temperature (Figure 4 a). In comparison, rM4 only retained 14 % activity under the same conditions (Figure 4 a). The significantly enhanced storage stability of M6 and CyM6 facilitates their utilization at ambient temperature over longer process time.

**Figure 4:**
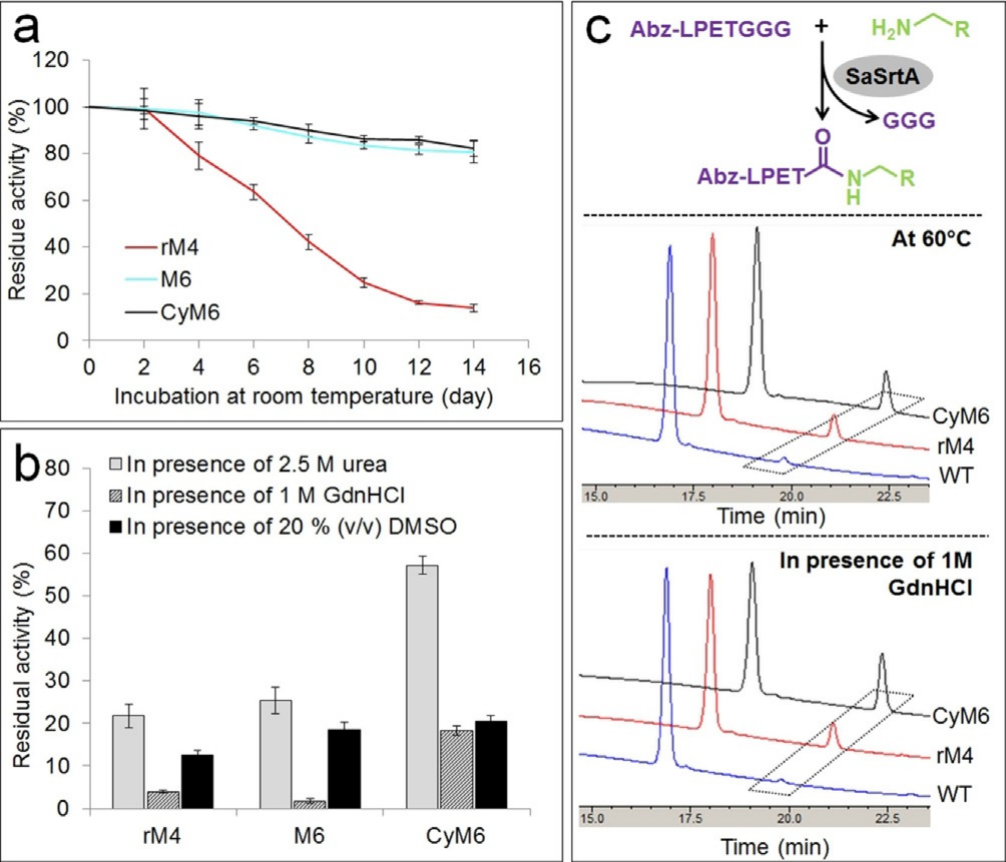
a) Storage stabilities of SaSrtA variants at room temperature. b) Stability of SaSrtA variants in presence of 1 m GdnHCl, 2.5 m urea or 20 % (v/v) DMSO. c) Sortagging of Abz‐LPETGGG peptide and primary amine nucleophile (tyramine) at high temperature (60 °C) and in presence of GdnHCl. The synthesized Abz‐LPETGGG‐tyramine is highlighted in diamond frame.

Resistance of the variants rM4, M6 and CyM6 against denaturants (guanidinium chloride (GdnHCl) and urea) and organic solvent (dimethyl sulfoxide (DMSO)) was investigated. The variant CyM6 showed the highest catalytic activities (vs. M6 and rM4) under all investigated conditions (Figure S10). Specifically, CyM6 gained 8.8‐, 4.5‐ and 2.9‐fold activity in presence of 1 m GdnHCl, 2.5 m urea and 20 % (v/v) DMSO, respectively, when compared to rM4 (Figure S10). Regarding stabilities, CyM6 showed 4.6 and 2.6‐fold resistance (vs. rM4) in 1 m GdnHCl and 2.5 m urea, respectively (Figure 4 b). The obtained resistance of CyM6 is in good agreement with previous studies in which enhanced resistance (4.5‐fold) of cyclized SaSrtA WT in GdnHCl and urea was observed.[^31^, ^32^]

To further evaluate the performance of CyM6 in sortagging reactions, the conjugation of a LPETG tagged peptide (Abz‐LPETGGG, Abz: 2‐aminobenzoyl) and primary amine nucleophile (tyramine) was conducted at 60 °C, in presence of 1 m GdnHCl, or 2.5 m urea (Figure 4 c). The formation of the peptide‐amine conjugate (Abz‐LPET‐tyramine) was confirmed by MALDI‐MS analysis (Figure S12) and quantified via high performance liquid chromatography (HPLC, Figure S11). After 20 min sortagging, rM4, M6, and CyM6 catalyzed clearly visible production of an Abz‐LPET‐tyramine conjugate while only negligible signals were observed for WT under all selected conditions (Figure 4 c/S11). CyM6 showed the highest activities under all conditions (1.9‐fold at 60 °C and 2.6‐fold in presence of 1 m GdnHCl) when compared to the parent rM4 (Figure 4 c/S11).

In summary, we established and validated a P450 BM3 reconstitution assay for its suitability in high‐throughput screening systems for SaSrtA. Engineering of highly active SaSrtA rM4 in the β6/β7 loop identified two key beneficial substitutions (R159N and K162P) for enhanced thermal stability. The key substitutions were recombined in the variant M6 resulting in increased thermal stability (ΔT_m_=+6.0 °C, T_m_: melting temperature) when compared to the parent rM4. Head‐to‐tail backbone cyclization of M6 yielded a cyclized variant CyM6 which retained full specific activity (99 % vs. M6) and further gained thermal stability (ΔT_m_=+7.5 °C vs. parent rM4). CyM6 retained 83 % (vs. 14 % of the parent rM4) activity after fourteen days of storage at room temperature. Additionally, CyM6 showed improved activity (up to 8.8‐fold vs. rM4), resistance (up to 4.6‐fold vs. rM4) and facilitated higher production (up to 2.6‐fold vs. rM4) of sortagged conjugates under thermal and denaturants stress. In essence, we generated robust SaSrtA variants with improved stability, which facilitate sortagging reactions in presence of denaturants, organic solvent over significantly enhanced thermal/storage stability. The latter advances sortagging reactions for synthetically attractive applications, such as continuous protein labelling/functionalization in flow‐based systems and large scale macro‐cyclization of pharmaceutical peptides.

## Conflict of interest

The authors declare no conflict of interest.

## Supporting information

As a service to our authors and readers, this journal provides supporting information supplied by the authors. Such materials are peer reviewed and may be re‐organized for online delivery, but are not copy‐edited or typeset. Technical support issues arising from supporting information (other than missing files) should be addressed to the authors.

Supplementary
