# Supplementary material for: Enhancing Robustness of Sortase A by Loop Engineering and Backbone Cyclization
Source: Chemistry. 2020 Aug 18;26(60):13568–72. doi: 10.1002/chem.202002740 (PMC7693181; doi:10.1002/chem.202002740)
Supplement: Supplementary file 1 — Supplementary [file CHEM-26-13568-s001.pdf]

Chemistry–A European Journal

Supporting Information

Enhancing Robustness of Sortase A by Loop Engineering and Backbone Cyclization

Zhi Zou,[a, b] Diana M. Mate,[b, c] Maximilian Nöth,[a, b] Felix Jakob,[a, b] and Ulrich Schwaneberg*[a, b]

Table of Contents

Material and Methods ... 1

Development of P450 BM3 reconstitution high-throughput assays for screening of sortase A .. 1

Gene construction of P450 BM3 his-heme-LPETGGGRR and GGG-his-reductase ... 1

Production of SaSrtA, P450 BM3 his-heme-LPETGGGRR and P450 BM3 GGG-his-reductase ... 2

SaSrtA mediated splicing of P450 BM3 his-heme-LPETG domain and GGG-his-reductase domain ... 3

P450 BM3 reconstitution for high-throughput screening of sortase A ... 4

Validation of P450 BM3 reconstitution for high-throughput screening of SaSrtA ... 5

Engineering of β6/β7 loop of SaSrtA for improved thermal stability ... 5

Single site saturation mutagenesis (SSM) libraries generation on β6/β7 loop ... 5

Screening of SSM libraries using P450 BM3 reconstitution assay ... 6

Recombination of beneficial substitutions ... 6

Head to tail cyclization of SaSrtA M6 variant ... 7

Production of AAA-his-SaSrtA M6-LPELAK-StrepII and his-SpSrtA M2- StrepII ... 7

Isolation of SaSrtA CyM6 from the sortagging mixture ... 7

Identification and characterization of SaSrtA CyM6 ... 8

Determination of the melting temperature of SaSrtAs ... 9

Resistance profiles of SaSrtA in presence of chaotropic agents and organic solvent ... 9

Storage stability of CyM6 ... 9

Profiles of SaSrtAs in conjugated product formation under denaturing conditions ... 10

List of tables ... 12

List of figures ... 15

Appendix ... 27

Protein sequence of SaSrtA variants ... 27

References ... 29

Material and Methods

Chemical reagents and solvents with analytical grade or higher purity were purchased from Sigma-Aldrich (Hamburg, Germany), AppliChem (Darmstadt, Germany), Carl Roth (Karlsruhe, Germany). Peptides Abz-LPETGK-Dnp-NH_2 (97.8%) and Abz-LPETGGG-COOH were purchased from Bachem (Bubendorf, Switzerland). Tyramine (99%) is purchased from Sigma-Aldrich. Enzymes used in polymerase chain reactions (PCRs) were all purchased from New England Biolabs (Frankfurt, Germany) or Fermentas (St. Leon-Rot, Germany). DNA oligos (primers) used in PCRs were purchased from Eurofins MWG Operon.

Development of P450 BM3 reconstitution high-throughput assays for screening of sortase A

Gene construction of P450 BM3 his-heme-LPETGGGRR and GGG-his-reductase

The reported P450 BM3 DM1 gene in pALXtreme plasmid[1] was used the template for the PCR construction of P450 BM3 with sequence of 462-468 replaced by a LPETGGG motif (S462L/T463P/E464E/Q465T/S466G/A467G /K468G)). Primers *Fw lpetggg 462-468* and *Rev lpetggg 462-468* were used (**Table S1**). The generated gene of P450 BM3 with the LPETGGG (462-468) motif was used as the template to construct the heme-LPETGGGRR domain or the GGG-reductase domain. For the PCRs, primers *Fw heme-lpetgggrr*, *Rev heme-lpetgggrr, Fw ggg-reductase* and *Rev ggg-reducatse* were used, respectively (**Table S1**). In the last step, a hexa histidine (His_6) gene sequence was inserted into the N-terminal of P450 BM3 heme-LPETGGGRR gene and GGG-reductase domain gene (after GGG motif). For PCRs, primers *Fw ggg-his-reductase*, *Rev ggg-his-reductase*, *Fw his-heme-lpetgggrr* and *Rev his-heme-lpetgggrr* were used (**Table S1**).

PCR solutions (25 μL) consist of 10 ng plasmid template, 10 mM dNTP mix, 50 uM of each primer (forward primer (*Fw*) and reverse primer (*Rev*), and 2.5 U Q5 DNA polymerase. PCR products were incubated by Dpn I (2.5 U, 37°C, overnight) and subsequently carried out a heat inactivation (80°C for 20 min) of the enzymes. PCR products were then transformed into *E.coli* BL-21 lac^{IQ} competent cells.

Production of SaSrtA, P450 BM3 his-heme-LPETGGGRR and P450 BM3 GGG-his-reductase

The expression (in shaking flask) and purification of SaSrtAs (WT and rM4 (P94S/D160N/D165A/K196T)) were implemented as previously reported.[2]

Expression of P450 BM3 LPETGGG (462-468), P450 BM3 his-heme-LPETGGGRR and P450 BM3 GGG-his-reductase were performed in shaking flask. In detail, Precultures (10 mL LB media, 50 μg/mL kanamycin) were inoculated from glycerol stocks and incubated (200 rpm, 16 h, 37°C, 70% humidity Multitron II Infors shaker). The main culture (1 L flask, 200 mL TB media, 50 μg/mL kanamycin, 1 mM tracing element solution) was inoculated with 2 mL pre-culture in Multitron II Infors shaker for ~3 h (200 rpm, 37°C, 70% humidity). At an OD600 of ~0.8 the main culture was induced (0.2 mM IPTG, 1 mM delta-aminolevulinic acid (ALA), and 1mM thiamine) and followed with incubation (200 rpm, 48 h, 20°C, 70% humidity). The expressed cells were harvested by centrifugation (3220 g, 30 min, 4°C, Eppendorf centrifuge 5810 R). The pellet of cells was stored at -20°C until for usage.

Cell pellet (one gram) was first suspended with lysozyme solution (10 mL, 0.5 mg/mL lysozyme, and 10 mM $CaCl_2$; 50 mM, Tris-HCl, pH 8.0) and incubated (30 min, 4°C, 600 rpm) and then sonicated on ice (60% amplitude, 24 cycles, 5 seconds per cycle, intervals 20 seconds). After centrifugation (3220 g, 1 h, 4°C) the supernatant and pellet were used for SDS-PAGE

analysis (**Figure S1a**). The clear supernatants of P450 BM3 his-heme-LPETGGGRR and P450 BM3 GGG-his-reductase were used for the purification (**Figure S1b**) or sortase-mediated P450 BM3 reconstitution high throughput assays in **Figure S4**.

The purification of P450 BM3 his-heme-LPETGGGRR and P450 BM3 GGG-his-reductase were conducted by using his-tag based purification. Purification was conducted by Protino Ni-IDA 2000 packed columns (Macherey-Nagel GmbH & Co. KG, Düren, Germany) using the protocol as the manufacture provided. Purified P450 BM3 his-heme-LPETGGGRR or P450 BM3 GGG-his-reductase fragments were pooled and further desalted with Amicon ultra-15 centrifugal filter units (10 kDa cut-off, Merck Millipore Ltd, Tullagreen,IRL). The purity of samples was analyzed by SDS-PAGE (11% gel, **Figure S1 b**).

SaSrtA mediated splicing of P450 BM3 his-heme-LPETG domain and GGG-his-reductase domain

The sortase-mediated splicing of P450 BM3 heme and reductase domains was firstly performed with purified samples. In short, His-heme-LPETGGGRR (10 μM), GGG-his-reductase (30 μM) and SrtA (5 μM WT or rM4 variants) were mixed in buffer B (5 mM $CaCl_2$, 50 mM Tris-HCl pH 8.0) and followed with an incubation (25 °C, 800 rpm, 35 min). Three controls were performed in alignment. Buffer A (50 mM Tris-HCl, pH 8.0) instead of His-heme-LPETGGGRR, GGG-his-reductase and SrtA was supplemented in control 1, 2 and 3, respectively (**Figure 1b**).

After incubation, 30 μL sortagged sample was transferred into black flat MTP and mixed with 60 μL buffer A (50 mM Tris-HCl pH 8.0). The substrate BCCE (2 μL, 2 mM in DMSO) was supplemented into the MTP and followed with incubation 25 °C, 800 rpm, 5 min). The assay was initiated by adding NADPH (8 μL, 6.25 mM). The fluorescence is constantly recorded (Tecan

infinite 1000Pro plate reader, λ_{exc} = 400 nm; λ_{em} = 440 nm, gain = 100; 2 min). Activity of reconstituted P450 BM3 was calculated as the slope of increased fluorescence (**Figure 1c**). Further evaluation of the reconstituted P450 BM3 was carried out by SDS-PAGE analysis (11% gel, **Figure 1b/S2**).

P450 BM3 reconstitution for high-throughput screening of sortase A

Thermal stability of SaSrtA WT and rM4 was investigated. The Abz-LPETGK-Dnp based fluorescence resonance energy transfer (FRET) assay was used to measure the activity of SaSrtA.[3] In the first step, purified SaSrtA were pipetted into a 250 µL PCR eppi tube and heated by gradient temperature (35 to 60 °C) for 1 h. In the second step, the Abz-LPETGK-Dnp based FRET assay was initiated by supplementing 2µM heated SaSrtA into the reaction mixture (100µL: 50 µM Abz-LPETGK-Dnp, 5 mM glycine-glycine-glycine, 5 mM $CaCl_2$, 150 mM NaCl, 50mM Tris/HCl pH 8.0). The gain of fluorescence was continuously recorded (λ_{exc} = 320 nm; λ_{em} = 420 nm, 5 min, room temperature, gain = 100, Tecan infinite 1000 PRO plate reader). Activity (slope of the fluorescence in the linear range, RFU/s) was calculated. The residual activity is defined as the ratio of obtained activity divided the activity at 35 °C. Data is given in **Figure S3**.

The **protocol** of P450 BM3 reconstitution for high-throughput screening of SaSrtA is illustrated in **Figure S4**. SaSrtA variants were both screened with and or without heating of incubation (55°C, 1h). The **protocol** is consisted of four main steps: In *Step 1* SaSrtA cell free lysate (35 µL) was transferred from the expression MTP (V-type 96-well MTP) to 96-well PCR-type MTP and subsequently incubated (55°C, 1h) in PCR machine (Eppendorf, Vapo protect) using a method (4°C for 1 min, temperature is slowly increased to 55 °C, temperature is slowly decreased to 4°C); In *Step 2,* the heated lysate (30 µL) is incubated (25 °C, 800 rpm, 35 min) with his-heme-LPETGGGRR (30 µL) and GGG-his-reductase (30 µL) in the black flat 96-well MTP; In *Step 3,*

the BCCE based fluorogenic assay[1] is initiated by supplementing NADPH (8 μL, 6.25 mM) and the fluorescence is constantly recorded (Tecan infinite 1000Pro plate reader, λ_{exc} = 400 nm; λ_{em} = 440 nm, gain = 100).

Validation of P450 BM3 reconstitution for high-throughput screening of SaSrtA

The validation of P450 BM3 reconstitution assay was performed with SaSrtA rM4 variant[2,4] for one MTP (96 colonies) using the protocol as above described. The background signal of the screening assay was performed by control experiments in which the empty vector cell instead SaSrtA rM4 cell free lysate was employed. The coefficient of variations (CoV) of 96 colonies screening were calculated (**Figure S5)**.

Engineering of β6/β7 loop of SaSrtA for improved thermal stability

Single site saturation mutagenesis (SSM) libraries generation on β6/β7 loop

SSM libraries at positions of 159, 161, 162, 163, 164, 166, 167, 168, 169, 170 and 172 libraries were generated, individually. Site 160 and 165 are beneficial positions for enhanced activity,[4] site 171 is crucial for calcium binding,[5,6] therefore they were not selected for SSM generation. SaSrtA rM4 was used as the template for SSM. Primers used in PCRs are list in **Table S2**. PCR solutions (50 μL) consist of 20 ng plasmid template, 10 mM dNTP mix, 50 μM of each primer (forward primer (*Fw*) and reverse primer (*Rev*), and 2.5 U Q5 DNA polymerase. PCR products were incubated by Dpn I (5 U, 37°C, overnight) and subsequently carried out a heat inactivation (80°C for 20 min). PCR products were then transformed into *E.coli* BL-21(DE3) competent cells. Clones of each single-site SSM library were transferred into two V-type 96-well polystyrene microtiter plate. Culture cultivation, expression and cell-free lysate preparation SaSrtA library variants in MTPs were performed as previously reported.[2]

Screening of SSM libraries using P450 BM3 reconstitution assay

SaSrtA SSM libraries (SSM 159, 161, 162, 163, 164, 166, 167, 168, 169, 170 and 172) were screening by using the P450 domain resconstitution assay. Variants showed improved activity (≥ 1.25 fold *vs* SaSrtA rM4) were selected for rescreening (four replicates each variant), individually. After rescreening, variants showed improved activity (≥ 1.5 fold *vs* SaSrtA rM4) were sequenced. Five variants (R159N, R159T, K162N, K162P, and K172L) with substitutions at three sites (R159, K162 and Q172) were identified (**Figure 2a**).

Recombination of beneficial substitutions

Site directed mutagenesis (SDM) was employed to recombine the identified beneficial substitutions. In a brief, the plasmid contains the gene of SaSrtA rM4-R159N was used as the gene template, primer *Fw SDM K162P* and *Rev SDM K162P* (**Table S3**) was used in the PCR to generate the variant SaSrtA rM4-R159N/K162P (M6), which renamed as M6. The plasmid of M6 was further used as the template to incorporate the third mutation Q172 using primer *Fw SDM Q172L* and *Rev SDM Q172L* (**Table S3**). The generated the variant SaSrtA rM4-R159N/K162P/Q172L was renamed as M7.

The recombined variants were expression in flask and purified with his-tag based chromatography as aforementioned. The Abz-LPETGK-Dnp based FRET assay was used to measure the activity of the purified samples. Firstly, purified SaSrtAs were pipetted into a 250μL PCR eppi tube and heated by the PCR machine at 55°C for 1 h using the protocol as described above. In the second step, the Abz-LPETGK-Dnp based FRET assay was initiated by supplementing 2 μM heated or non-heated (as controls) SaSrtA into the reaction mixture (100μL: 50 μM Abz-LPETGK-Dnp, 5 mM glycine-glycine-glycine, 5 mM $CaCl_2$, 150 mM NaCl, 50mM Tris/HCl pH 8.0). The gain of fluorescence was continuously recorded (λ_{exc} = 320 nm; λ_{em} = 420

nm, 5 min, room temperature, gain = 100). Activity (slope of the fluorescence in the linear range, RFU/s) is given in **Figure S6**.

Head to tail cyclization of SaSrtA M6 variant

Production of AAA-his-SaSrtA M6-LPELAK-Strep Ⅱ and his-SpSrtA M2- Strep Ⅱ

Construction of AAA-his-SaSrtA M6-LPELAK-StrepⅡ was conducted in three steps. In the first step, the gene of his-SaSrtA M6 was used as template to construct AAA-his-SaSrtA M6 where primers *Fw AAA-his-SaSrtA* and *Rev AAA-his-SaSrtA* were used (**Table S3**). In the next step, the generated gene of AAA-his-SaSrtA M6 was used as template to construct AAA-his-SaSrtA M6-LPELAK in which primers *Fw AAA-his-SaSrtA-LPELAK* and *Rev AAA-his-SaSrtA-LPELAK* were used (**Table S3**). In the last step, the gene sequence of Strep Ⅱwas included by PCR in which *Fw AAA-his-SaSrtA-LPELAK-Strep Ⅱ* and *Rev-AAA-his-SaSrtA-LPELAK-Strep Ⅱ* were used (**Table S3**). The gene of *Streptococcus pyogenes* sortase A variant (V206I/E215A SpSrtA M2) was the template to construct *his-SpSrtA-Strep Ⅱ* in which primers *Fw-his-SpSrtA-Strep Ⅱ* and *Rev-his-SpSrtA-Strep Ⅱ* were used (**Table S3**). The plasmids with target genes were transformed into *E. coli* BL-21 (DE3) competent cell. Expression and purification of purification of AAA-his-SaSrtA M6-LPELAK-Strep Ⅱ and his-SpSrtA M2-Strep Ⅱ were performed as described for SaSrtA.[2]

Isolation of SaSrtA CyM6 from the sortagging mixture

The cyclization of AAA-his-SaSrtA M6-LPELAK-StrepⅡwas performed by using SpSrtA M2 recognizing an orthogonal -LPELA/ -AAA sortagging pair. In brief, 15 μM AAA-his-SaSrtA M6-LPELAK-StrepⅡ, 5μM his-SpSrtA M2-StrepⅡ, 150 mM NaCl in 10 mL Tris/HCl pH 8.0

buffer were incubated (25°C，800 rpm, 6 h). After incubation 30 μL was pipetted put and mixed with 10 μL 4xSDS loading buffer for SDS-PAGE analysis (line 3 in **Figure S7b**). The rest reaction solution were firstly diluted with 12 mL buffer (Tris/HCl, 50 mM pH 8.0) and collected in a 25 mL springe. The diluted reaction solution was manually and slowly injected into the StrepTrap HP column (GE health Science, Heidelberg, Germany, **Figure S7a**). The flow through were collected**.** In the next step, the StrepTrap HP column was washed with 30 mL buffer (Tris/HCl, 50 mM pH 8.0) and solution was collected**.** In the last step, Strep-trapped protein in the StrepTrap HP column was eluted with eluting solution (2.5 mM desthiobiotin, 30 mL, Tris/HCl, 50 mM pH 8.0) and collected the eluted solution. The collected solutions were desalted and concentrated to 3 mL using Amicon ultra-15 centrifugal filter units (3 kDa cut-off, Merck Millipore Ltd) and analyzed with SDS-PAGE (**Figure S7b** line 4, 5 and 6), respectively.

Identification and characterization of SaSrtA CyM6

To further confirm the formation of SaSrtA CyM6, the purified SaSrtA CyM6 was subjected for Matrix-Assisted Laser Desorption/Ionization Mass Spectrum (MALDI-MS). In detail, 50 microliter purified SaSrtA CyM6 was diluted with 2 mL distill water and supplemented in the Amicon ultra-2 mL centrifugal filter units (10 kDa cut-off, Merck Millipore Ltd) and centrifuged (3220 g, 1 h, 4°C, Eppendorf centrifuge 5810 R). After centrifugation, flow-through was discarded and the same desalting step was repeated. Desalted sample was collected and the volume was adjusted to 50 μL. The desalted SaSrtA CyM6 (2 μL) was then mixed with super DHB and crystallized on ground steel for MALDI-MS analysis (Bruker UTX MALDI-TOF/TOF MS/MS, **Figure 3c**). Activity of SaSrtA CyM6 was determined using the Abz-LPETGK-Dnp based FRET assay using a same protocol as aforementioned in analysis of purified SaSrtA. Data is given in **Figure S8**.

Determination of the melting temperature of SaSrtAs

Circular dichroism (CD) spectra of SaSrtAs were performed using the JASCO J-1100 spectrometer (JASCO Deutschland GmbH, Pfungstadt, Germany). Six micromolar SaSrtA (115 μg/mL in 350 μL distill water) was analyzed in a 400 μL cuvette at room temperature. The CD spectrum of SaSrtA WT, rM4, M6 and CyM6 is shown in **Figure 3d**. All the SaSrtAs showed similar CD spectrum profile and a sharp turn of CD signal at 201 nm was observed.

To obtain the melting temperature of SaSrtA and variants, CD spect was ra of SaSrtA were recorded in gradient increased temperatures (25 to 80°C, at a heating rate of 1°C min^{-1}). The CD signal at 201 nm (**Figure S9**) plotted against the temperature and apparent melting temperatures (T_m) were obtained using the OriginPro 9.0 (OriginPro Software, OriginLabs, **Figure 3e**).

Resistance profiles of SaSrtA in presence of chaotropic agents and organic solvent

Activity of SaSrtA CyM6 in presence of 2.5 M urea, 1 M guanidinium chloride (GdnHCl) or 20% (v/v) DMSO were measured using the Abz-LPETGK-Dnp based FRET assay. In brief, the FRET reaction mixture (100 μL: 50 μM Abz-LPETGK-Dnp, 5 mM glycine-glycine-glycine, 5 mM $CaCl_2$, 150 mM NaCl, 2.5 M urea (or 1M GdnHCl or 20 μL DMSO), Tris/HCl pH 8.0). After incubation (room temperature, 800 rpm, 3 min), FRET reaction was initiated by supplementing SaSrtA (2.5 μL, 20 μM). Fluorescence was recorded and activity was obtained using the protocol as aforementioned (**Figure S10**).

Storage stability of CyM6

Storage stability of CyM6 at room temperature was investigated. In brief, SaSrtA CyM6 (20 μL, 20 μM) was incubated at room temperature for up to fourteen days. For each two days, 2 μL

SaSrtA was pipetted out and diluted in 18 μL Tris/HCl (50mM, pH 8.0). Ten microliter diluted SaSrtA sample was supplemented to initiate the sortaggings (90 μL, 50 mM Abz-LPETGK-Dnp, 5 mM Gly-Gly-Gly, 5 mM CaCl2, 150 mM NaCl, 50 mM Tris/HCl pH 8.0). Fluorescence of sortagging samples was recorded as above described. Storage stability was calculated as the ratio of SaSrtA activity after x days storage at room temperature divided SaSrtA activity without storage at room temperature (**Figure 4a**).

Profiles of SaSrtAs in conjugated product formation under denaturing conditions

To evaluate the performance of SaSrtA CyM6 sortagged product formation under different denaturing conditions, sortagging between Abz-LPETGGG and amine (tyramine) was performed.

In brief, sortaggings (100 μL, 1 mM Abz-LPETGGG, 5 mM tyramine, 5 μM SaSrtA (WT; rM4, M6 or CyM6), 5 mM CaCl2, 150 mM NaCl, 50 mM Tris/HCl pH 8.0) was incubated at 60°C or in presence of 1 M GdnHCl for 20 min. The reactions were quenched by adding HCl (1 M; 50 μL) and followed by centrifugation (room temperature, 20 min, 1200 g).

Ten microliter HCl quenched sample was analyzed via a reversed phase high performance liquid chromatography (RP-HPLC, C18 column, 4.6x150 mM, 5 μM, Agilent, Düren, Germany). A gradient of 10 to 95% acetonitrile/0.1% TFA (0 to 25 min) was used. Absorbance of tyramine, Abz-LPETGGG and Abz-LPET-tyramine conjugate were recorded at 254 nm (**Figure 4c** and **Figure S11**).

One hundred microliter HCl quenched sample was transferred in to Amicon ultra-2 mL centrifugal filter units with 10 kDa cut-off (Merck Millipore Ltd) for desalting. Three milliliter distilled water was supplemented in the filter unit and centrifuged (3220 g, 1 h, 4°C, Eppendorf

centrifuge 5810 R). The flow-through was discarded and the same step of desalting was repeated. Desalted sample was collected and adjusted the volume (with distilled water) to 50 μL. The desalted sample (2 μL) was mixed with super DHB (2 μL) and crystallized on the target (ground steel) for Matrix-Assisted Laser Desorption/Ionization Mass Spectrum (MALDI-MS, Bruker UTX MALDI-TOF/TOF MS/MS, **Figure S12**).

List of tables

Table S1. List of primers in the gene construction of his-heme-LPETGGGRR and GGG-his-reductase.

**Primer Name**	**Sequence 5'-3'**
Fw lpetggg 462-468	CTCCCTGAAACGGGTGGCGGGAAAGTACGCAAAAAGGCAG
Rev lpetggg 462-468	CCCGCCACCCGTTTCAGGGAGAGGTGAAGGAATACCGCC
Fw heme-lpetgggrr	GGGTGGCGGGCGCCGCTAAGAATTCGAGCTCCGTCG
Rev heme-lpetgggrr	CTCGAATTCTTAGCGGCGCCCGCCACCCGTTTCAGG
Fw ggg-reductase	CTGGGCAGCATGGGTGGCGGGAAAGTACGCAAAAAG
Rev ggg-reducatse	CTTTCCCGCCACCCATGCTGCCCAGGGTATATCTCC
Fw his-heme-lpetgggrr	CATCATCATCATCATCACGGCACAATTAAAGAAATGCCTC
Rev his-heme-lpetgggrr	GTGATGATGATGATGATGCATGGTATATCTCCTTCTTAAAG
Fw ggg-his-reductase	CATCATCATCATCATCACAAAGTACGCAAAAAGGCAGAAAAC
Rev ggg-his-reductase	GTGATGATGATGATGATGCCCGCCACCCATGCTGCCCAGG

Table S2. List of primers used for the site-saturation mutagenesis (N means A, C, G or T, M means A or C and K means G or T).

**Primer Name**	**Sequence 5’-3’**
Fw SSM R159	GACAAGTATANNKAATGTTAAGCC
Rev SSM R159	GGCTTAACATTMNNTATACTTGTC
Fw SSM V161	GTATAAGAAATNNKAAGCCAACAG
Rev SSM V161	CTGTTGGCTTMNNATTTCTTATAC
Fw SSM K162	ATAAGAAATGTTNNKCCAACAGCTG
Rev SSM K162	CAGCTGTTGGMNNAACATTTCTTAT
Fw SSM P163	GAAATGTTAAGNNKACAGCTGTAGA
Rev SSM P163	TCTACAGCTGTMNNCTTAACATTTC
Fw SSMD164	GTTAAGCCANNKGCTGTAGAAG
Rev SSM D164	CTTCTACAGCMNNTGGCTTAAC
Fw SSM V166	CCAACAGCTNNKGAAGTTCTAG
Rev SSM V166	CTAGAACTTCMNNAGCTGTTGG
Fw SSM E167	CAACAGCTGTANNKGTTCTAGATG
Rev SSM E167	CATCTAGAACMNNTACAGCTGTTG
Fw SSM V168	CAGCTGTAGAANNKCTAGATGAAC
Rev SSM V168	GTTCATCTAGMNNTTCTACAGCTG
Fw SSM L169	GCTGTAGAAGTTNNKGATGAACAAAAAG
Rev SSM L169	CTTTTTGTTCATCMNNAACTTCTACAGC
Fw SSM D170	GAAGTTCTANNKGAACAAAAAG
Rev SSM D170	CTTTTTGTTCMNNTAGAACTTC
Fw SSM Q172	GTTCTAGATGAANNKAAAGGTAAAG
Rev SSM Q172	CTTTACCTTTMNNTTCATCTAGAAC

Table S3. List of primers used for recombination.

**Primer Name**	**Sequence 5'-3'**
Fw SDM K162P	AATAATGTTCCTCCAACAGCT
Rev SDM K162P	AGCTGTTGGAGGAACATTATT
Fw SDM Q172L	GTTCTAGATGAACTGAAAGGTAAAG
Rev SDM Q172L	CTTTACCTTTCAGTTCATCTAGAAC
Fw AAA-his-SaSrtA	ATGGCGGCAGCGAGCACGCATCATCATCATC
Rev AAA-his-SaSrtA	GCTCGCTGCCGCCATGGTATATCTCCTTC
Fw AAA-his-SaSrtA-LPELAK	CTGCCGGAACTGGCGAAACACCACCACCAC
Rev AAA-his-SaSrtA-LPELAK	TTTCGCGGTTTCCGGCAGTTTGACTTCTGTAG
Fw AAA-his-SaSrtA-LPELAK-Strep II	TGGAGCCATCCGCAGTTCGAAAAGTGAGATCCGGCTGCTAACAAAGCC
Rev AAA-his-SaSrtA-LPELAK-Strep II	CTTTTCGAACTGCGGATGGCTCCATTTCGCCAGTTCCGGCAGTTT G
Fw-his-SpSrtA- Strep II	TGGAGCCATCCGCAGTTCGAAAAGTAACTCGAGCA
Rev-his-SpSrtA- Strep II	CTTTTCGAACTGCGGATGGCTCCATGTAGACACCT

List of figures

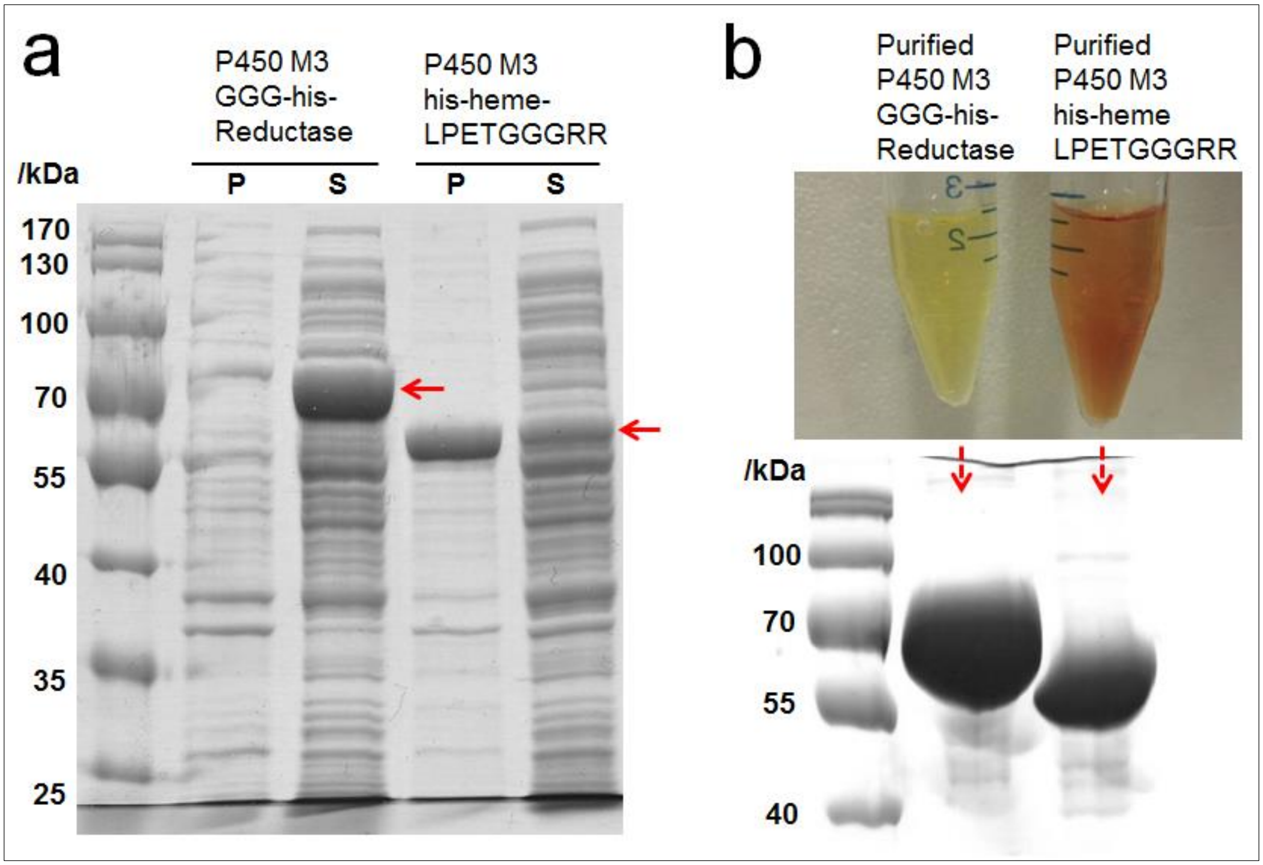

Figure S1. Production of P450 BM3 his-heme-LPETGGGRR and GGG-his-reductase domains. **a**) Expression of P450 BM3 his-heme-LPETGGGRR and GGG-his-reductase domains. The expected molecular weight of P450 BM3 his-heme-LPETGGGRR and GGG-his-reductase are 54.4 and 65.4 kDa, respectively. The soluble expressed domains are marked by red arrow (P: pellet of cell lysis; S: supernatant of cell lysis). **b**) Purification of P450 BM3 his-heme-LPETGGGRR and GGG-his-reductase domains using his-tag based chromatography (upper: photos of purified samples; bottom: SDS-PAGE of purified samples).

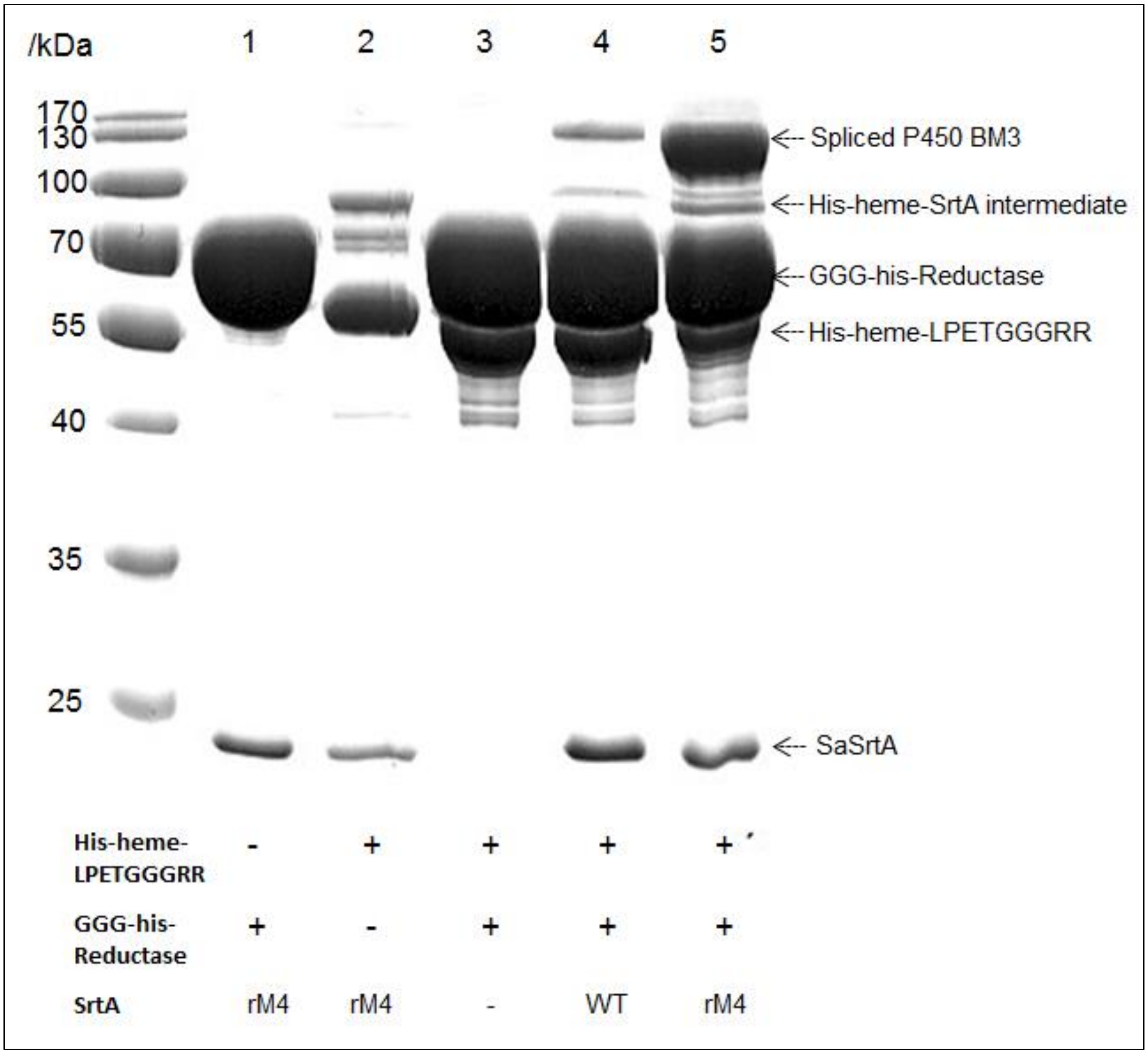

Figure S2. SaSrtA mediated splicing of P450 BM3 his-heme-LPETG and GGG-his-reductase domains (full size picture of Figure **1b**). Domains splicing (reaction solution: 10 μM his-heme-LPETGGGRR, 30 μM GGG-his-reductase and 5 μM, SaSrtA, Tris/HCl pH 8.0) was performed by incubation (25°C, 800 rpm, 35 min). The generation of reconstituted P450 BM3 (in sample 4 and 5) was confirmed by SDS-PAGE.

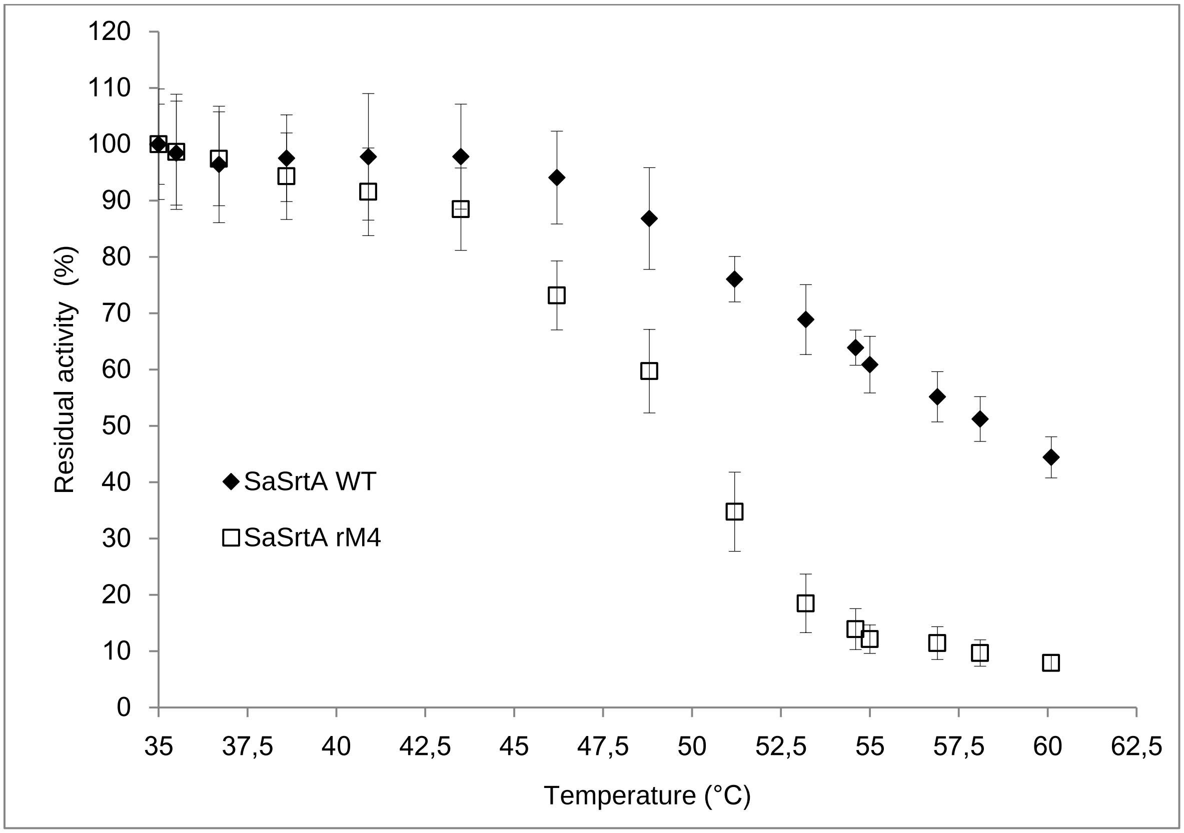

Figure S3. Residual activities of SaSrtA (WT and rM4) after incubation for 1 h at gradient temperatures.

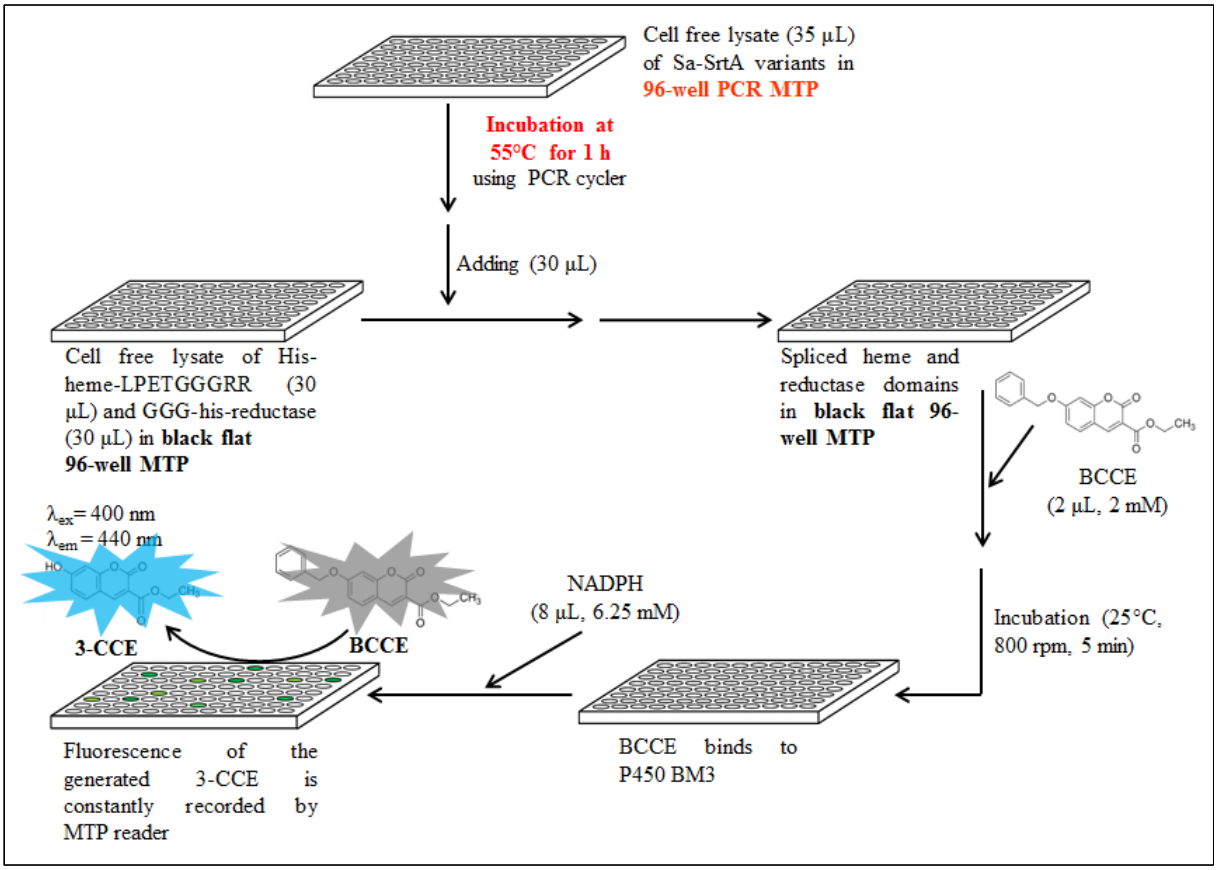

Figure S4. Schematic representations of P450 BM3 reconstitution assay for high throughput screening of SaSrtA of improved transpeptidase activity in 96-well MTP format. BCCE: 7-benzoxy-3-carboxycoumarin ethyl ester; 3-CCE: 3-carboxycoumarin ethyl ester.

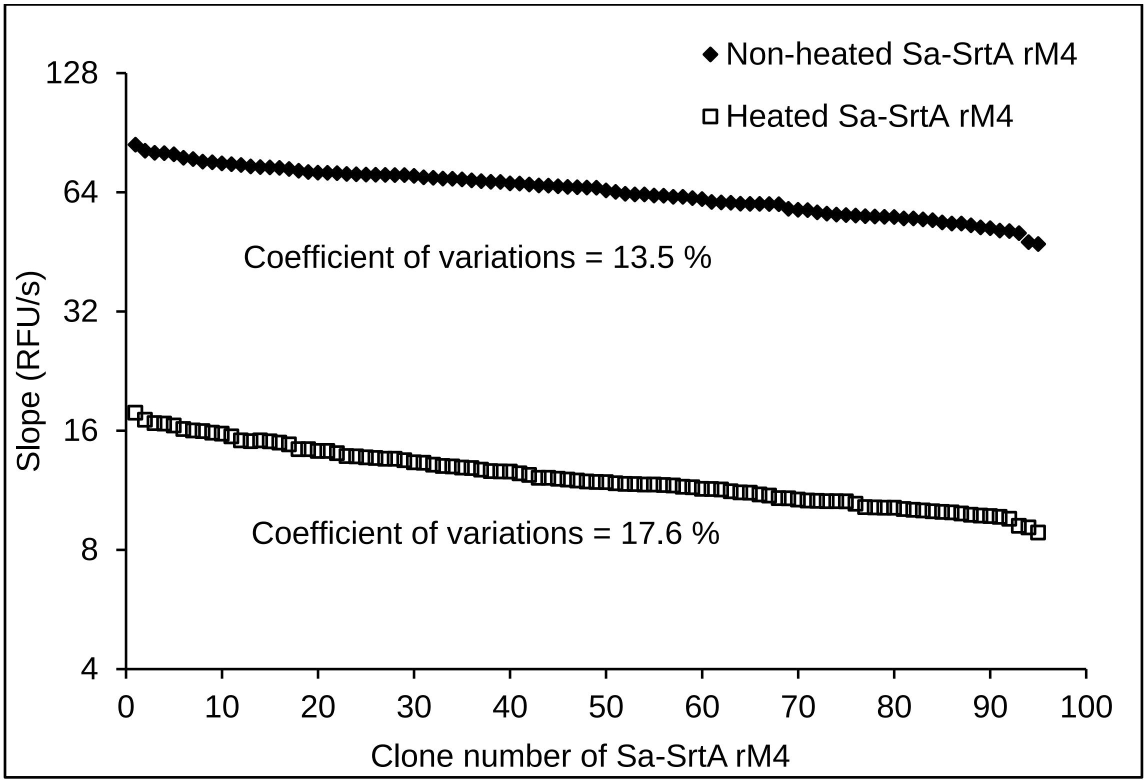

Figure S5. Coefficient of variation of P450 BM3 reconstitution assay in screening of 96 SaSrtA rM4 colonies using thermal incubated (55°C, 1 h) and non-incubated SaSrtA rM4 cell lysate.

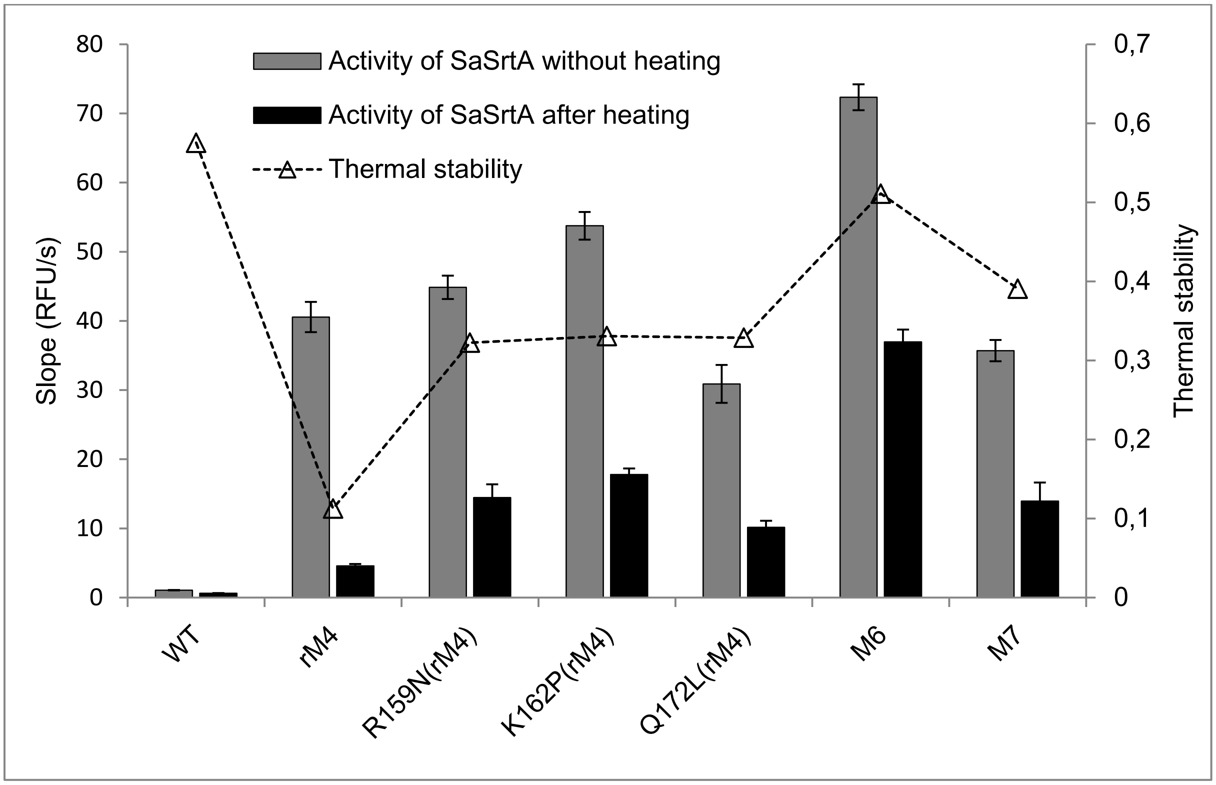

Figure S6 Activity of purified SaSrtA samples (WT and variants) in the Abz-LPETGK-Dnp based FRET assay. Activities of variants were measured with or without thermal incubation (40 μL, 50 μM SaSrtA, at 55°C for 1 h). FRET reaction contains (0.05 mM Abz-LPETGK-Dnp, 5 mM glycine-glycine-glycine, 1 μM SaSrtA, 5 mM CaCl2, 150 mM NaCl, Tris/HCl pH 8.0). Thermal stability is defined as the ratio of SaSrtA activity after thermal incubation divided by the activity without thermal incubation.

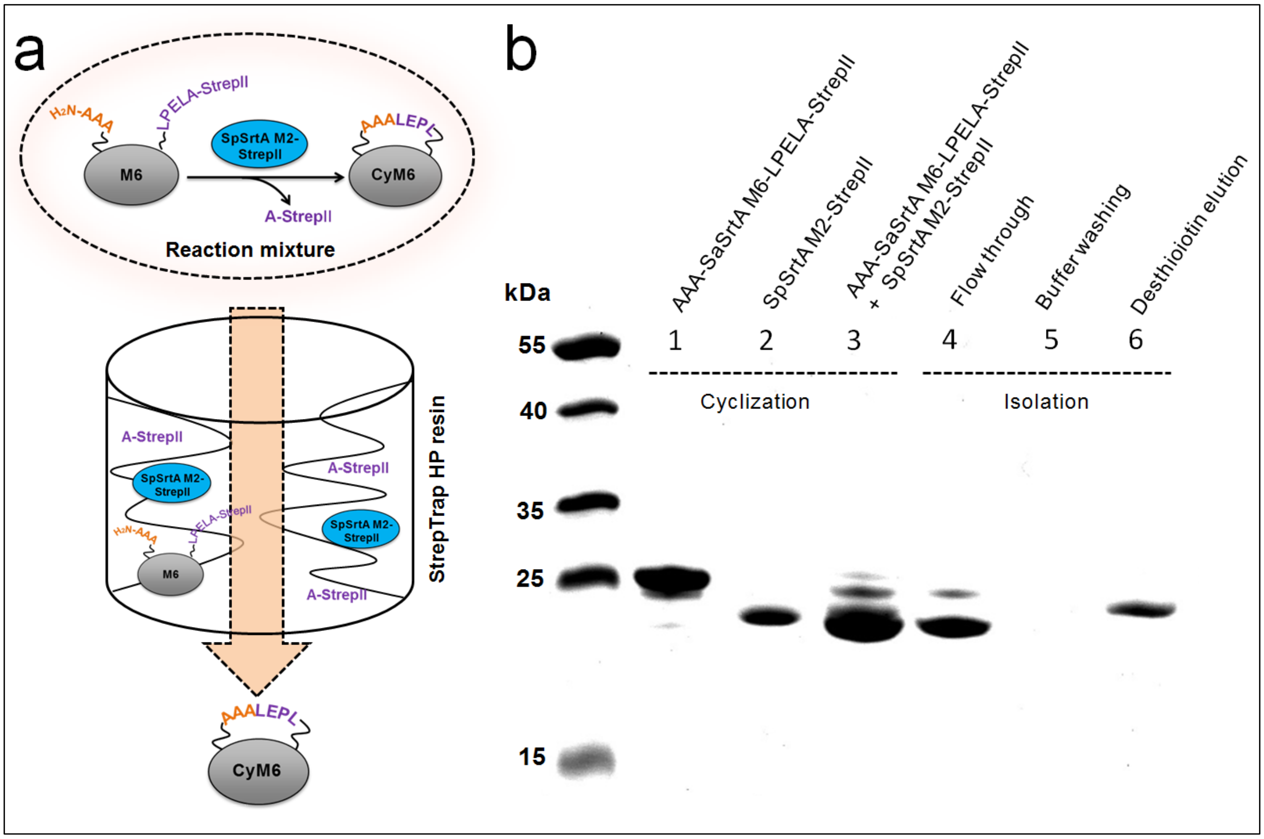

Figure S7. Separation of SaSrtA CyM6 from the sortagging mixture. **a**) Schematic representation of a StrepTrap resin based chromatography protocol for isolation of SaSrtA CyM6. Components with Strep II (AAA-SaSrtA M6-LPELA-Strep II, SpSrtA M2- Strep II) were captured by the StrepTrap resin. The cyclized SaSrtA CyM6 (CyM6) without Strep II was collected from the flow through. **b**) SDS-PAGE (tricine gel 16%) analysis of processing samples in the purification of SaSrtA CyM6. The sample in **lane 3** was loaded into the StrepTrap resin and samples from isolation processes were showed as in lane 4, 5 and 6.

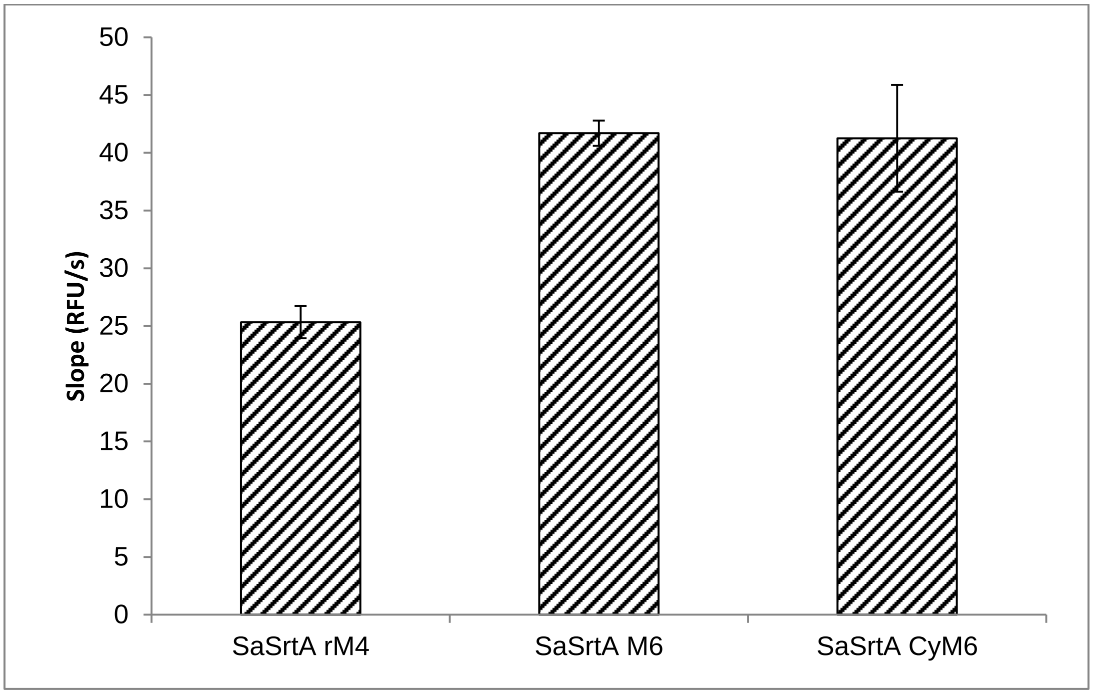

Figure S8. Activity of SaSrtA CyM6 determined by Abz-LPETGK-Dnp based FRET assay. The reaction (100 μL: 50 μM Abz-LPETGK-Dnp, 5 mM glycine-glycine-glycine, 5 mM $CaCl_2$, 150 mM NaCl, 50mM Tris/HCl pH 8.0) was initiated by supplementing 0.5 μM purified SaSrtA.

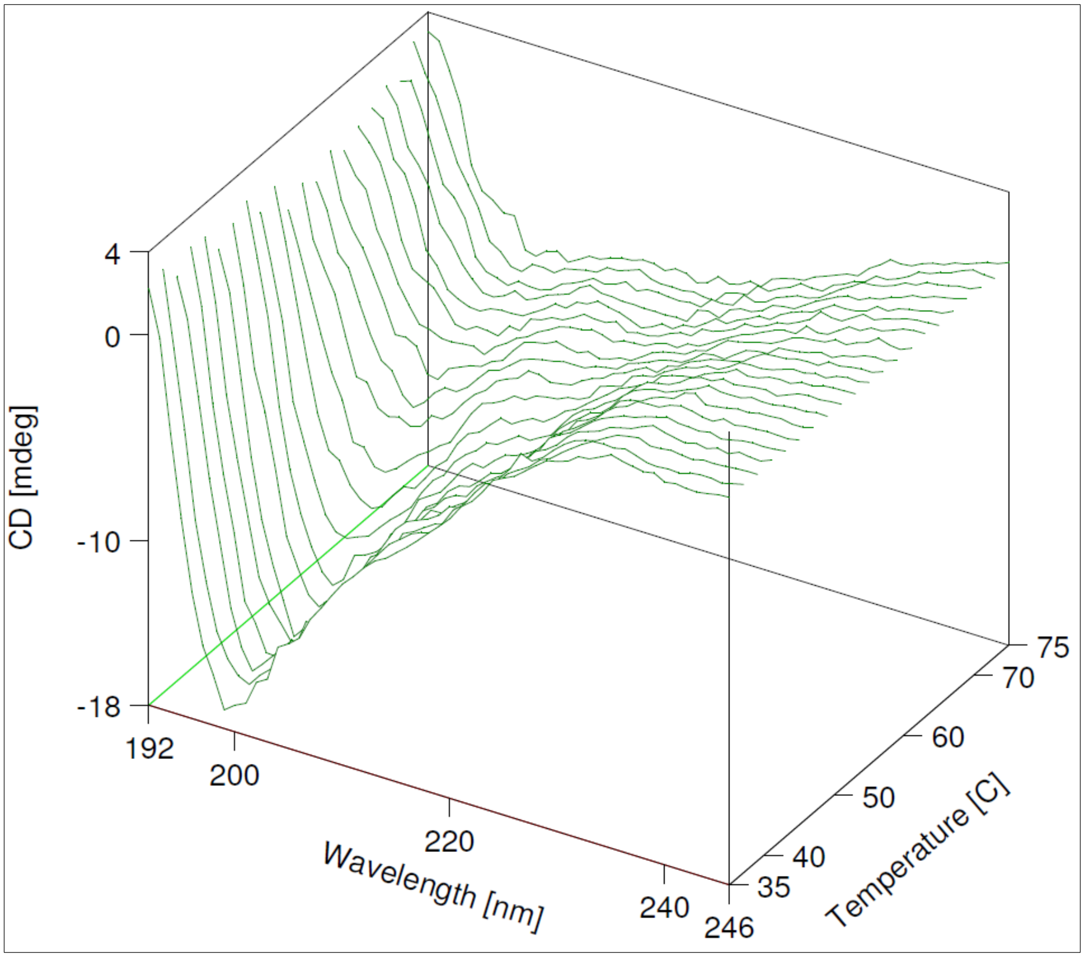

Figure S9. Circular dichroism (CD) spectra of SpSrtA M6 under gradient temperatures (35 to 75℃). Sharp turns of CD signal at 201 nm were observed. Wavelength scan spectra from 240 to 193 nm were recorded in triplets and averaged.

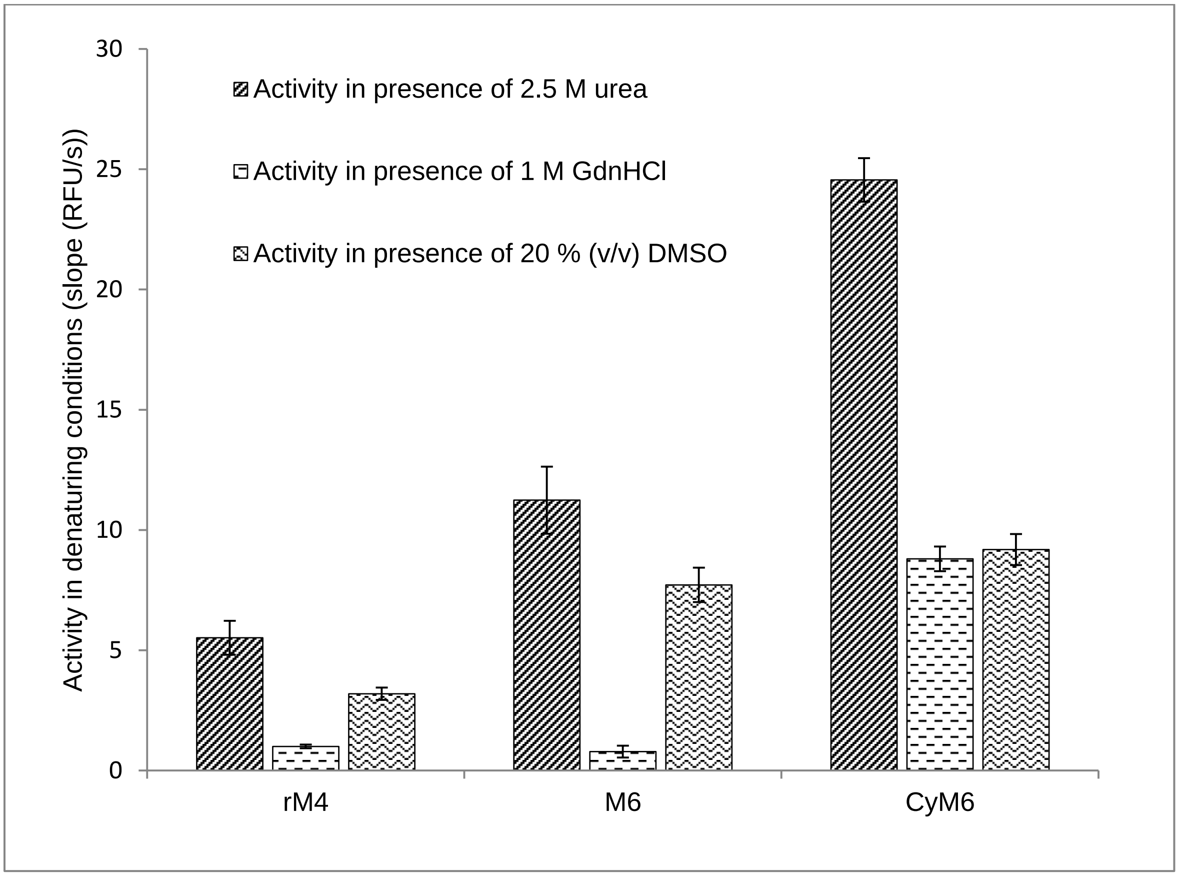

Figure S10. Activity profiles of SaSrtA variants in present of 2.5 M urea, 1 M GdnHCl or 20% (v/v) DMSO. The reactions (100 µL: 50 µM Abz-LPETGK-Dnp, 5 mM glycine-glycine-glycine, 5 mM $CaCl_2$, 150 mM NaCl, 50mM Tris/HCl pH 8.0, 2.5 M urea or 1M GdnHCl or 20% (v/v) DMSO) was initiated by supplementing 0.5 µM purified SaSrtA.

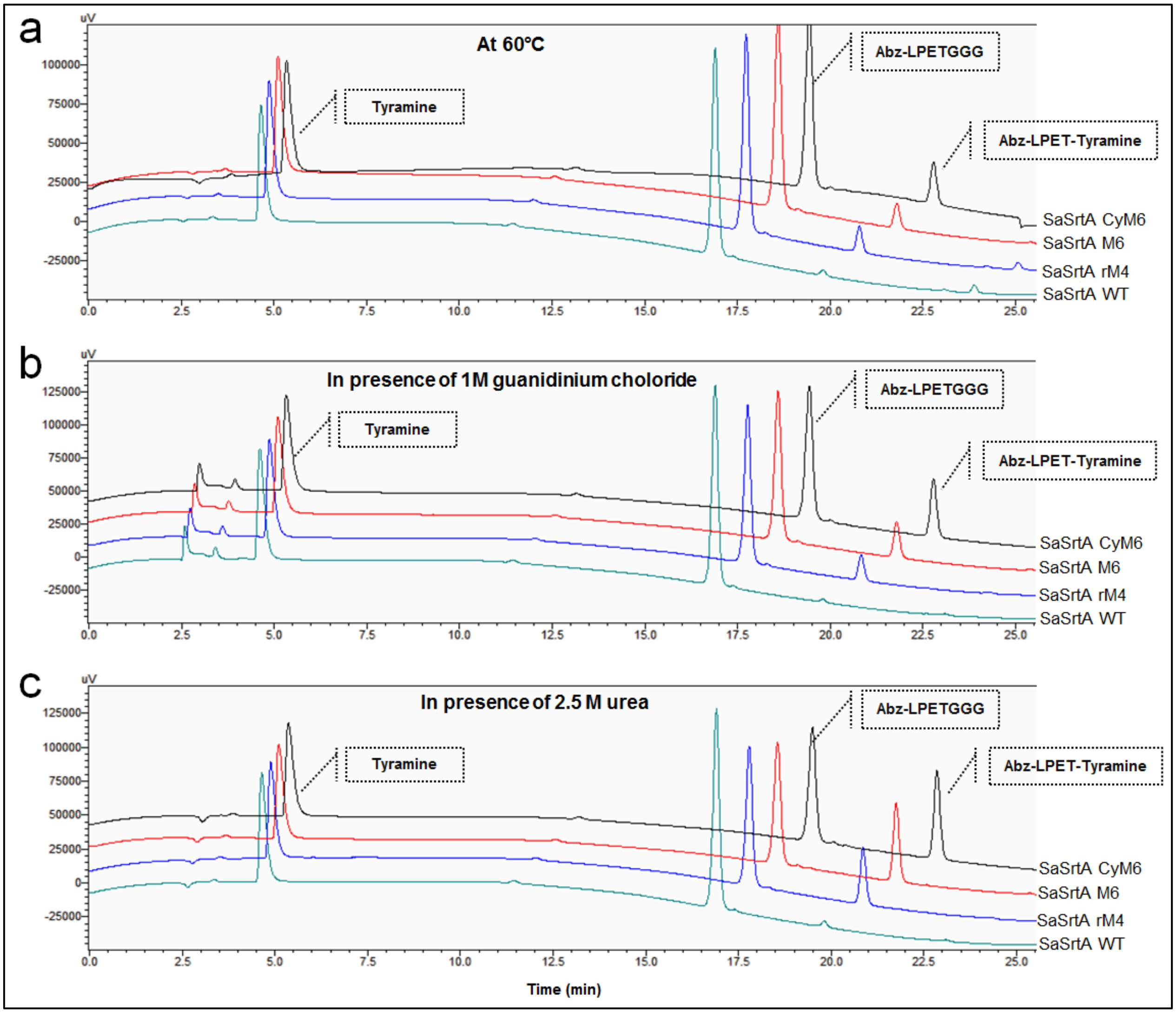

Figure S11. HPLC tracing (at 254nm, full size picture of **Figure 3b**) of sortaggings between Abz-LPETGGG and tyramine at 60℃ (**a**), in presence of 1 M GdnHCl (**b**), or 2.5 M urea (**c**).

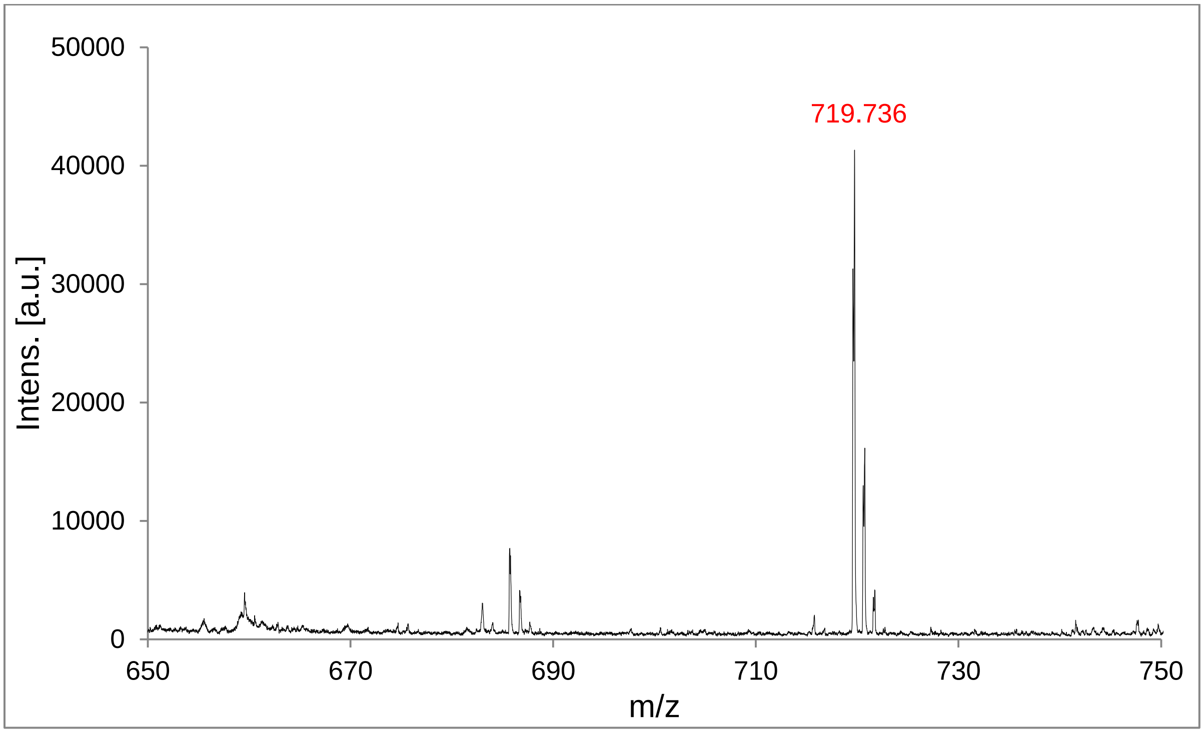

Figure S12. MALDI-TOF-MS of sortagging between Abz-LPETGGG and tyramine at 60 ℃ (as an example to confirm the formation of Abz-LPET-tyramine conjugate). The expected molecular weight of **Abz-LPET-tyramine** conjugate is 696.32 Da. The mass peak at 719.736 Da indicates the peak of Abz-LPET-tyramine including a Na^+.

Appendix

Protein sequence of SaSrtA variants

Amino acid sequence SaSrtA rM4

MGSSHHHHHHSSGLVPRGSHMQAKPQIPKDKSKVAGYIEIPDADIKEPVYPGPATSEQLNRGVSF

AEENESLDDQNISIAGHTFIDRPNYQFTNLKAAKKGSMVYFKVGNETRKYKMTSIRNVKPTAVEV

LDEQKGKDKQLTLITCDDYNEKTGVWETRKIFVATEVK

Amino acid sequence SaSrtA rM4-R159N

MGSSHHHHHHSSGLVPRGSHMQAKPQIPKDKSKVAGYIEIPDADIKEPVYPGPATSEQLNRGVSF

AEENESLDDQNISIAGHTFIDRPNYQFTNLKAAKKGSMVYFKVGNETRKYKMTSI**N**NVKPTAVEV

LDEQKGKDKQLTLITCDDYNEKTGVWETRKIFVATEVK

Amino acid sequence SaSrtA rM4-K162P

MGSSHHHHHHSSGLVPRGSHMQAKPQIPKDKSKVAGYIEIPDADIKEPVYPGPATSEQLNRGVSF

AEENESLDDQNISIAGHTFIDRPNYQFTNLKAAKKGSMVYFKVGNETRKYKMTSIRNV**P**PTAVEV

LDEQKGKDKQLTLITCDDYNEKTGVWETRKIFVATEVK

Amino acid sequence SaSrtA M6

M**AAA**GSSHHHHHHSSGLVPRGSHMQAKPQIPKDKSKVAGYIEIPDADIKEPVYPGPATSEQLNRG

VSFAEENESLDDQNISIAGHTFIDRPNYQFTNLKAAKKGSMVYFKVGNETRKYKMTSI**N**NV**P**PTA

VEVLDEQKGKDKQLTLITCDDYNEKTGVWETRKIFVATEVK**LPELAKWSHPQFEK**

Amino acid sequence SaSrtA M7

M**AAA**GSSHHHHHHSSGLVPRGSHMQAKPQIPKDKSKVAGYIEIPDADIKEPVYPGPATSEQLNRG

VSFAEENESLDDQNISIAGHTFIDRPNYQFTNLKAAKKGSMVYFKVGNETRKYKMTSI**N**NV**P**PTA

VEVLDE**L**KGKDKQLTLITCDDYNEKTGVWETRKIFVATEVK**LPETAKWSHPQFEK**

Amino acid sequence SpSrtA M2-StrepⅡ

MGHHHHHHSSGLVPRGSVLQAQMAAQQLPVIGGIAIPELGINLPIFKGLGNTELIYGAGTMKEEQ

VMGGENNYSLASHHIFGITGSSQMLFSPLERAQNGMSIYLTDKEKIYEYIIKDVFTVAPERVDVI

DDTAGLKEVTLITCTDIEATARIIVKGELKTEYDFDKAPADVLKAFNHSYNQVST**WSHPQFEK**

References

[1] A. J. Ruff, A. Dennig, G. Wirtz, M. Blanusa, U. Schwaneberg, *ACS Catal.* **2012**, *2*, 2724–2728.

[2] Z. Zou, H. Alibiglou, D. M. Mate, M. D. Davari, F. Jakob, U. Schwaneberg, *Chem. Commun.* **2018**, *54*, 11467–11470.

[3] R. G. Kruger, P. Dostal, D. G. McCafferty, *Anal. Biochem.* **2004**, *326*, 42–48.

[4] I. Chen, B. M. Dorr, D. R. Liu, *Proc. Natl. Acad. Sci. U. S. A.* **2011**, *108*, 11399–11404.

[5] M. T. Naik, N. Suree, U. Ilangovan, C. K. Liew, W. Thieu, D. O. Campbell, J. J. Clemens, M. E. Jung, R. T. Clubb, *J. Biol. Chem.* **2006**, *281*, 1817–1826.

[6] N. Suree, C. K. Liew, V. A. Villareal, W. Thieu, E. A. Fadeev, J. J. Clemens, M. E. Jung, R. T. Clubb, *J. Biol. Chem.* **2009**, *284*, 24465–24477.
